# A simplified MyProstateScore2.0 for high-grade prostate cancer

**DOI:** 10.1177/18758592241308755

**Published:** 2025-03-20

**Authors:** Tiffany M Tang, Yuping Zhang, Ana M Kenney, Cassie Xie, Lanbo Xiao, Javed Siddiqui, Sudhir Srivastava, Martin G Sanda, John T Wei, Ziding Feng, Jeffrey J Tosoian, Yingye Zheng, Arul M Chinnaiyan, Bin Yu

**Affiliations:** 1 Department of Statistics, University of Michigan, Ann Arbor, MI, USA; 2 Department of Pathology, University of Michigan, Ann Arbor, MI, USA; 3 Michigan Center for Translational Pathology, University of Michigan, Ann Arbor, MI, USA; 4 Department of Statistics, University of California, Irvine, Irvine, CA, USA; 5 Department of Biostatistics, Fred Hutchinson Cancer Research Center, Seattle, WA, USA; 6 Division of Cancer Prevention, National Institutes of Health, Bethesda, MD, USA; 7 Department of Urology, Emory University, Atlanta, GA, USA; 8 Department of Urology, University of Michigan, Ann Arbor, MI, USA; 9 Department of Urology, Vanderbilt University Medical Center, Nashville, TN, USA; 10 Vanderbilt-Ingram Cancer Center, Nashville, TN, USA; 11 Howard Hughes Medical Institute, Chevy Chase, MD, USA; 12 Departments of Statistics and Electrical Engineering and Computer Science, University of California, Berkeley, CA, USA; 13 Center for Computational Biology, University of California, Berkeley, CA, USA

**Keywords:** prostate cancer, urine biomarker, PCS for veridical data science, machine learning, trustworthy AI

## Abstract

**Background:**

The limited diagnostic accuracy of prostate-specific antigen screening for prostate cancer (PCa) has prompted innovative solutions, such as the state-of-the-art 18-gene urine test for clinically-significant PCa (MyProstateScore2.0 (MPS2)).

**Objective:**

We aim to develop a non-invasive biomarker test, the simplified MPS2 (sMPS2), which achieves similar state-of-the-art accuracy as MPS2 for predicting high-grade PCa but requires substantially fewer genes than the 18-gene MPS2 to improve its accessibility for routine clinical care.

**Methods:**

We grounded the development of sMPS2 in the Predictability, Computability, and Stability (PCS) framework for veridical data science. Under this framework, we stress-tested the development of sMPS2 across various data preprocessing and modeling choices and developed a stability-driven PCS ranking procedure for selecting the most predictive and robust genes for use in sMPS2.

**Results:**

The final sMPS2 model consisted of 7 genes and achieved a 0.784 AUROC (95% confidence interval, 0.742–0.825) for predicting high-grade PCa on a blinded external validation cohort. This is only 2.3% lower than the 18-gene MPS2, which is similar in magnitude to the 1–2% in uncertainty induced by different data preprocessing choices.

**Conclusions:**

The 7-gene sMPS2 provides a unique opportunity to expand the reach and adoption of non-invasive PCa screening.

## Introduction

Prostate specific antigen (PSA) is the most widely used biomarker for early screening of prostate cancer, contributing to significant decline in prostate cancer (PCa) mortality.^1^ Despite the benefit, the low specificity of PSA due to its organ-specific rather than cancer-specific nature, can lead to overdiagnosis and overtreatment. To combat these challenges, tremendous effort has been made to develop non-invasive and more specific biomarker tests, especially to distinguish clinically-significant or high-grade PCa (i.e., Grade Group 2 PCa and higher) from dormant or low-grade PCa. In particular, the discovery of recurrent gene fusion *TMPRSS2:ERG* (*T2:ERG*) in prostate cancer^2^ led to the development of MyProstateScore test (MPS), which measures expression of prostate cancer antigen 3 (*PCA3*) and *T2:ERG* in clinical urine specimens^3^ and has been endorsed by the National Comprehensive Cancer Network for consideration prior to biopsy in men with elevated PSA.^4^

Since the development of MPS, high-throughput gene expression profiling has become readily available through RNA sequencing from large-scale cancer studies, such as The Cancer Genome Atlas (TCGA). Leveraging public and in-house RNA-seq data from PCa tumor and normal prostate, we previously nominated 54 biomarkers and established the second generation of MyProstateScore named MPS2, an 18-gene urine assay to predict high-grade PCa.^5^ The gene panel was validated on an independent or external cohort, achieving equivalent performance as in the development cohort (area under the receiver operating characteristic [AUROC] 0.81) and surpassing the performance of MPS (AUROC 0.74).

Given the previous concerns regarding PSA and possible overdiagnosis of indolent, low-grade prostate cancer, we stress the importance of demonstrating the robustness and generalizability of non-invasive biomarker tests such as MPS and MPS2. Notably, in any statistical or data science process, including those leading to the development of aforementioned biomarkers, there are numerous human judgment calls that must be made. These decisions include but are not limited to data preprocessing (e.g., how to impute missing values, how to handle outliers or erroneous measurements, how to filter of genes or handle correlated variables) and modeling choices (e.g., which models, which tuning parameters). When left unchecked, these necessary but often arbitrary choices introduce sources of uncertainty that are ignored in the traditional statistical uncertainty quantification, unknowingly alter downstream conclusions, and lead to poor generalizability in new patient cohorts.

Recently, the Predictability, Computability, and Stability (PCS) framework^6,7^ was developed to provide an overarching philosophical and practical framework for both stress-testing these choices and mitigating their unwanted impacts so as to bolster more reliable, trustworthy scientific conclusions. In short, the PCS framework advocates the need for transparent documentation of human judgment calls throughout the analysis pipeline and revolves around three core principles – *predictability* as a quantifiable assessment of whether the model sufficiently captures reality, *computability* as a necessary consideration in algorithmic design and data collection, and *stability* across data and model perturbations as a minimum requirement for veridical (trustworthy) science. These principles were originally motivated by extensive interdisciplinary research^8,9^ and have since led to numerous novel and extensively-validated scientific findings including epistatic mechanisms,^10,11^ stable biomarker discoveries,^12,13^ and interpretable clinical decision-making.^14,15^

In this work, our primary aim is to ease the translation of MPS2 into routine clinical care by substantially reducing the number of required genes in the test while preserving its high accuracy for predicting high-grade PCa. To this end, we leverage the PCS framework to perform a reliable, stability-driven selection of genes and distill the 18-gene MPS2 test into a simplified 7-gene MPS2 test (s^7^MPS2) while maintaining its high predictive power. Through this investigation, we demonstrate the accuracy and robustness of s^7^MPS2 as well as the original MPS2 test to various data preprocessing and modeling choices, thereby bolstering their trustworthiness and generalizability.

## Materials and methods

### Development cohort

The same development cohort used to build the original MPS2 models was used.^5^ Briefly, prebiopsy urine samples (first-catch urine following digital rectal examination) were prospectively collected at the University of Michigan from patients presenting for 12-core or greater prostate biopsy due to elevated PSA levels (3–10 ng/mL) from 2008 to 2020. A total of 761 samples were included in the final development cohort. We defer additional details to Tosoian et al. (2024).^5^

### External validation cohort

The external validation cohort was the same one used in the original MPS2 study.^5^ The cohort consisted of 743 patients in the prospective NCI EDRN PCA3 Evaluation Trial.^16^ This trial enrolled consecutive patients presenting for biopsy across 11 academic centers, primarily due to elevated PSA levels or abnormal digital rectal examination findings.

### Data preprocessing of gene expression data

Using qPCR profiling from OpenArray^™^, we measured gene expression in each urine sample via the cycle threshold (Ct), or the number of amplification cycles required for sample fluorescence to exceed the background level. Lower Ct values suggest higher gene expression. In this work, we focus our analysis on the 54 genes nominated in the MPS2 study.^5^ Then, because of the inevitably many different but equally-reasonable choices that could be made during the data preprocessing, we chose to preprocess this gene expression data using four different data preprocessing pipelines (detailed in Appendix A) and proceeded to evaluate the robustness of our model and conclusions across each of these data preprocessing pipelines in accordance with the PCS framework for veridical data science.^6,7^ This helps to ensure that the downstream scientific findings are not solely reliant on a particular data preprocessing decision.

### Predictor and outcome variables

Throughout this study, the set of predictor variables included the 54 nominated genes (from Section Data Preprocessing of Gene Expression Data) as well as available clinical variables that are generally associated with high-grade PCa (age, race, family history of prostate cancer, abnormal DRE, prior negative biopsy, and PSA).^17^ These variables were used to predict the primary outcome of interest – namely, high-grade PCa, defined as a dichotomous variable indicating whether the PCa is Grade Group 2 or higher. This is considered clinically significant PCa in this patient group (i.e., patients who should then undergo MRI or biopsy).

### Modeling choices

We considered many different statistical and/or machine learning models for predicting high-grade PCa. Namely, we considered common statistical or machine learning models such as ordinary logistic regression, logistic regression with *L*_*1*_ (LASSO) regularization,^18^ *L*_*2*_ (ridge) regularization,^19^ and combined *L*_*1*_ + *L*_*2*_ (elastic net) regularization,^20^ random forests (RF),^21^ gradient boosting decision trees,^22^ and RuleFit.^23^ We also investigated recently developed tree-based machine learning methods including random forest+ (RF+),^24^ a PCS-guided generalization of random forests which combines the strength of both linear models and nonlinear trees, and fast interpretable greedy-tree sums (FIGS),^25^ which grows a flexible but controllable number of shallow decision trees in summation. We focused primarily on these interpretable linear and tree-based models given our goal of identifying important genes for reliable biomarker development. Moreover, we note that tree-based machine learning models are often well-suited for biological tasks such as this, in part due to the resemblance between the thresholding behavior of decision trees and the on-off switch-like behavior commonly thought to govern genetic processes.^26^ Specific implementations of each model and their hyperparameters are detailed in Table 1.

### Model development: PCS ranking

**Figure 1.**
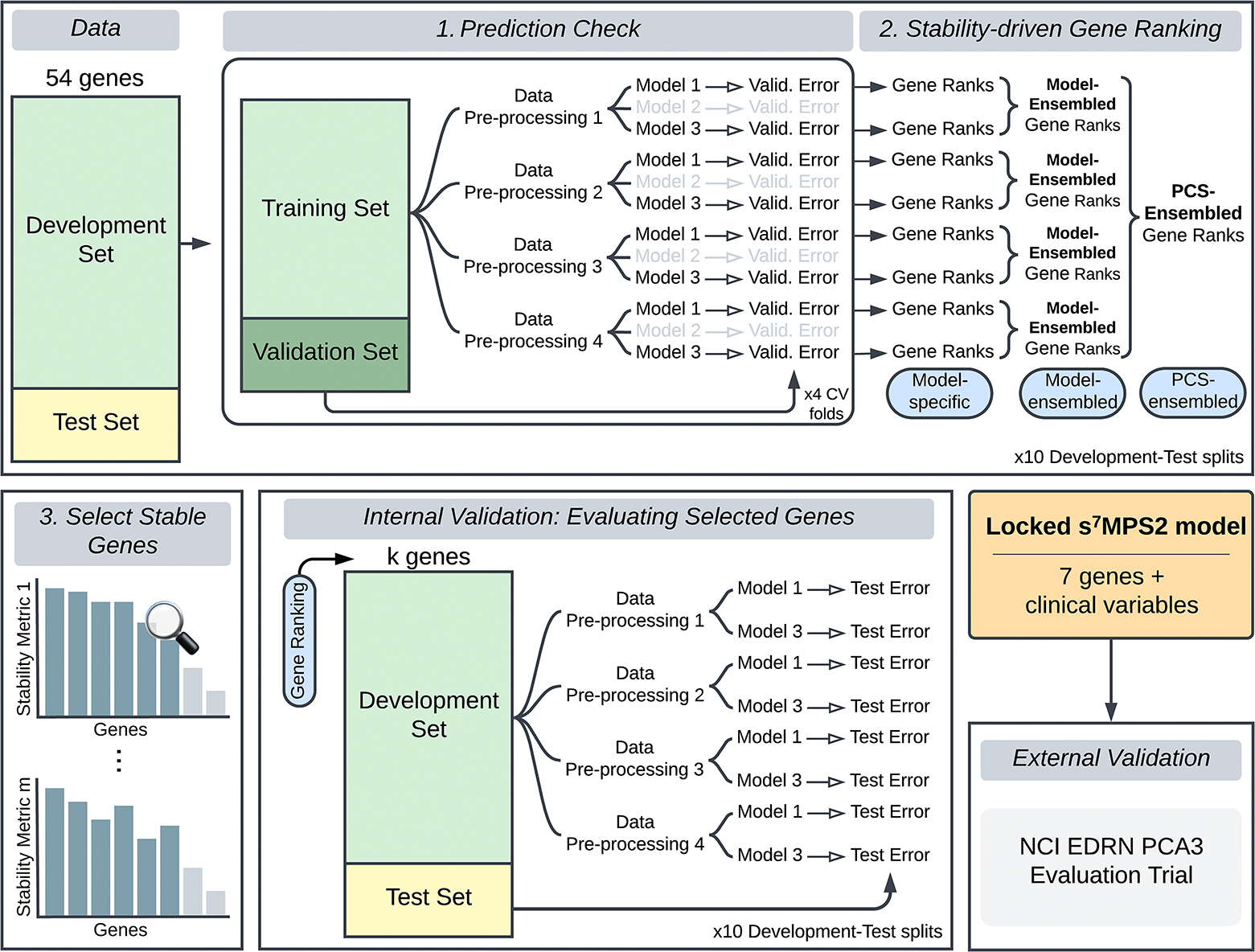
To develop the s^7^MPS2 model, we first split the Development cohort data into a Development and Test Set and leveraged the Development Set to (1) conduct a prediction check, where we evaluated the prediction performance for various machine learning models across four different data preprocessing pipelines, filtering out models with poor prediction performance, and (2) subsequently rank the most important genes across all data preprocessing pipelines and prediction-checked models. Only the most important and stable genes, measured according to multiple stability metrics (stage 3), were selected to be used in the final locked s^7^MPS2 model. To validate the quality and size of the gene panel used in s^7^MPS2, we performed an internal validation, examining the test prediction performance using alternative gene rankings and panel sizes (*k*). We lastly validated the final, locked s^7^MPS2 model on a blinded, external validation set using the NCI EDRN PCA3 Evaluation Trial.

Leveraging expression data from the 54 nominated genes and available clinical variables (age, race, family history of prostate cancer, abnormal DRE, prior negative biopsy, and PSA),^17^ we built the simplified MPS2 model (sMPS2) to predict high-grade PCa defined as grade group 2 or higher PCa. More specifically, using the Development Cohort data, we developed and applied the PCS ranking procedure, which consists of three main stages (Figure 1) – (1) a prediction check stage, where we evaluated model prediction performance, (2) a stability-driven gene ranking stage, where we ranked the importance of each gene using the models that have passed the prediction check in stage 1, and (3) a selection of stable genes stage, where we identified the most stably important genes for use in the final sMPS2 model. Each of these stages is heavily rooted in the PCS framework for veridical data science.^6,7^ This work builds upon similar ideas from the RF literature^9,10,11^ and extends them to different classes of methods. Further details regarding the PCS ranking can be found in Appendix B alongside a supplementary PCS documentation on GitHub with extensive justification for the many human judgment calls made throughout the data analysis pipeline (https://github.com/Yu-Group/sMPS2). We note that proper data splitting is a crucial ingredient to enable the generalizability of our developed model. We thus outline the data splitting procedure in the supplementary materials in Figure S1.

### Internal validation for evaluating selected genes

To evaluate the gene rankings from Stage 2 as well as the choice of the number of selected genes, we performed an internal validation using the test set from each of our 10 Development-Test splits (the same splits used in Stage 1). That is, for each Development-Test split, gene ranking from that given Development-Test split, and choice of *k* (*k* = 1, 2, 3, 4, 5, 6, 7, 10, 12, 15, 17, 20, 25, 30, 40, 54), we (a) took the top *k*-ranked genes and the available clinical features as covariates, (b) trained each prediction-checked model on each preprocessed Development set split, and (c) evaluated the prediction error (i.e., AUROC, AUPRC, and classification accuracy) on the test set. These errors were then averaged across the 10 Development-Test splits. We examined these averaged prediction errors across different choices of the gene panel size *k* and prediction models. Note that we also evaluated the different ways of obtaining the gene rankings (i.e., model-specific, model-ensembled, and PCS-ensembled).

### Final simplified MPS2 model (sMPS2)

The final covariate gene set in the simplified MPS2 model consisted of the 6 topmost important stable genes: *T2:ERG*, *SCHLAP1*, *OR51E2*, *TFF3*, *PCAT14*, and *PCA3*. Using these 6 genes and 6 clinical features (age, race, family history of prostate cancer, abnormal DRE, prior negative biopsy, and PSA) as covariates, we trained a logistic regression with *L*_*2*_ (ridge) regularization to predict high-grade PCa using the full Development Cohort dataset. This final trained model is referred to as the s^7^MPS2 model as it requires the measurement of 7 genes (i.e., the 6 genes used as covariates and the reference gene *KLK3* which is necessary for data preprocessing). Here, we used a logistic regression with *L*_*2*_ (ridge) regularization due to its strong performance in the internal validation assessment (Section Internal Validation for Evaluating Selected Genes). Since the inclusion of prostate volume in clinical models is well-known to improve the prediction of high-grade PCa,^27,28^ we also trained an analogous model, termed s^7^MPS2+, which includes all of the covariates in s^7^MPS2 plus prostate volume, for use when a patient’s prostate volume is readily available. For comparison, we also investigated and provided the external validation results for the s^8^MPS2 (and s^8^MPS2+) model, which is analogous to s^7^MPS2 (and s^7^MPS2+) but includes the top 7 most stable genes as covariates (*T2:ERG*, *SCHLAP1*, *OR51E2*, *TFF3*, *PCAT14*, *PCA3,* and *APOC1*). Finally, we calibrated the models to account for differences in outcome prevalence between the development and the validation cohorts following a logistic recalibration method^29^ as in Tosoian et al. (2024).^5^ These calibrated sMPS2 models were locked prior to external validation.

### Model validation on blinded, external cohort

We evaluated the final locked sMPS2 models on a blinded, external cohort (n = 743) from the NCI EDRN PCA3 Evaluation Trial^16^ described in Section External Validation Cohort. We importantly note that this external validation cohort data was only accessible by two investigators (C.X. and Y. Zheng), who performed the validation. The AUROC from the locked sMPS2 models were compared against MPS^3^ and MPS2.^5^ These AUROCs were then used to approximate the minimum test tradeoff (MTT) for an additional predictor.^30^ Specifically, the test tradeoff is defined as the minimum number of data collections per true positive to yield a positive net benefit.^30^ The MTT helps to formally quantify the improvement in risk prediction while accounting for the additional data collection cost of the 18-gene MPS2, compared to the smaller sMPS2 models. Moreover, we also computed a range of MTT corresponding to the range of mean AUROCs from different data preprocessing choices in order to shed light on how this important source of uncertainty might affect the MTT.

## Results

### Development of the simplified MyProstateScore 2.0 (sMPS2) model

Grounded by the PCS framework for veridical data science,^6^ we developed a stability-driven machine learning pipeline, termed PCS ranking, to build a robust and accurate risk score model of high-grade PCa using substantially fewer genes than MPS2. In this PCS-guided development pipeline, we rigorously assessed the accuracy and stability of modeling results across both data preprocessing and modeling pipelines in order to account for the inherent uncertainty arising from such human judgment calls and thus more faithfully capture the uncertainty and generalizability when deployed in reality.^6^ Briefly, the PCS ranking pipeline for sMPS2 consists of three main stages: (1) A prediction check stage, where we evaluated the prediction performance for a variety of machine learning models across four different data preprocessing pipelines and filtered out models with poor prediction performance; (2) A stability-driven gene ranking stage, where we ranked the importance of each gene according to both its magnitude of importance and the stability of its importances across model fits, data preprocessing pipelines, and data splits; and (3) A selection of stable genes stage, where we selected the set of most stable important genes for use in the final, locked sMPS2 model. Details regarding each step are provided in Section Model Development: PCS Ranking and Appendix B. Here, by identifying and focusing on the most important genes that were highly stable across both data preprocessing and modeling choices, we ensure that the final locked sMPS2 model is not solely a random artifact resulting from human analysis decisions, but rather a robust clinical risk model, which we will show to have highly predictive and generalizable performance (Sections Internal assessment and validation of the sMPS2 models and External Cohort Validation).

### Prediction check of machine learning models via cross validation

**Figure 2.**
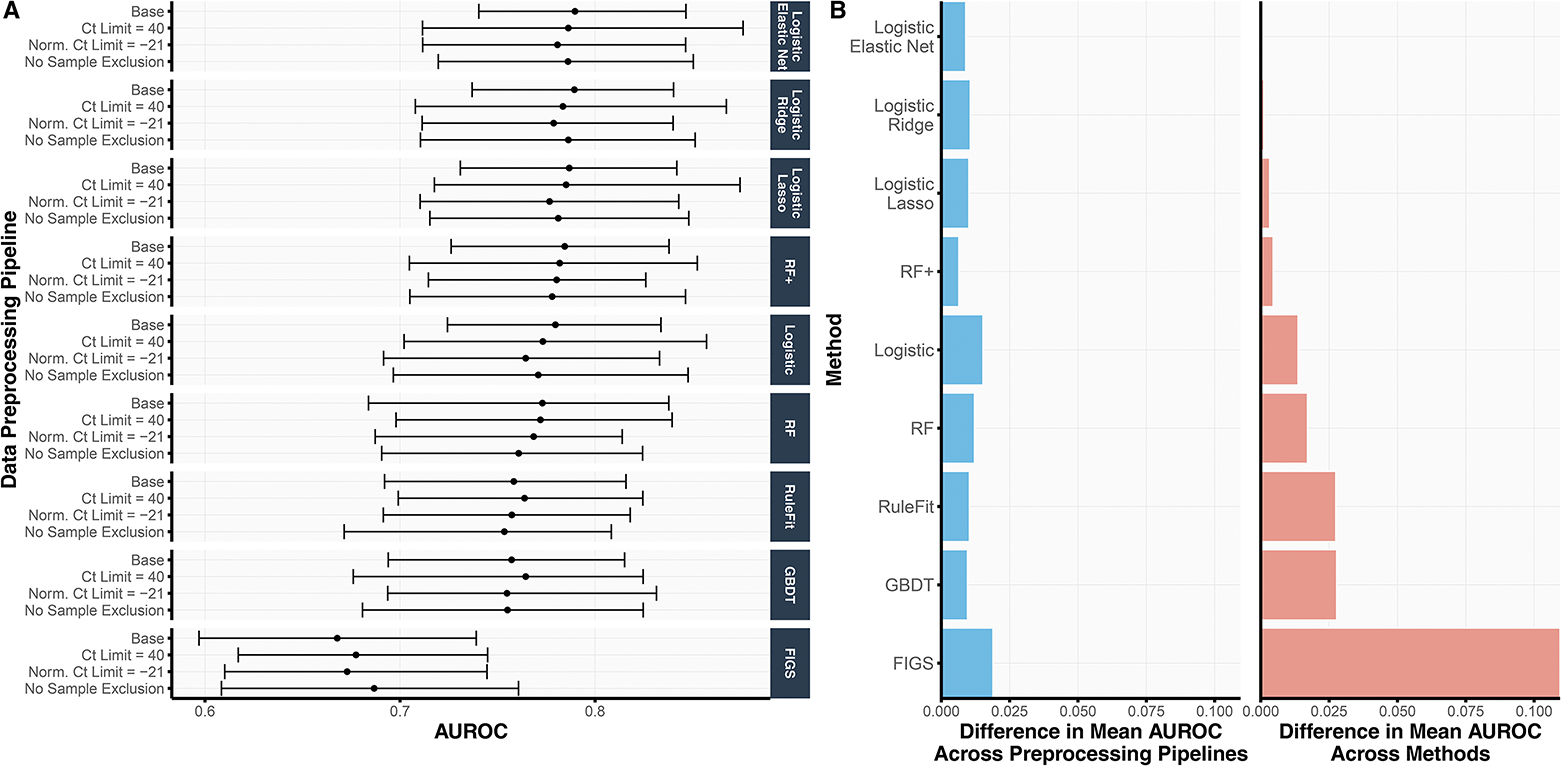
(A) For each choice of data preprocessing and prediction model, the validation AUROC, averaged across 4 CV folds and 10 repeated development-test splits, is shown. The error bars represent the inner 95% quantile range of the distribution of AUROCs. That is, across the 40 AUROCs, generated from 4-fold CV over 10 repeated Development-Test splits, the error bars correspond to the lower 2.5th and upper 97.5th quantiles of the distribution. (B) We compare the variation in AUROC across data preprocessing pipelines (left) and methods (right). In the left subplot, we show the range of mean AUROCs across the four data preprocessing pipelines for each method. In the right subplot, we show the difference between the mean AUROC from each method and the best performing method (i.e., logistic regression with the elastic net penalty) across all data preprocessing pipelines. The difference in AUROC across data preprocessing pipelines is substantially smaller than that across prediction methods, suggesting that the development pipeline and downstream findings are robust to data preprocessing choices.

As part of the prediction check stage in the development of sMPS2 (Figure 1), we assessed the cross-validation prediction performance for models trained with all 54 genes and available clinical variables across nine different machine learning models and four different preprocessing pipelines. We summarize the AUROC results from 4-fold CV repeated over 10 Development-Test splits in Figure 2 and provide the analogous AUPRC and classification accuracy results in Figure S2. As shown in Figure 2(A), the linear-based models (i.e., both regularized and unregularized logistic regression) tended to outperform the non-linear tree-based models (i.e., RF, GBDT, RuleFit, and FIGS), suggesting some smooth underlying structure in the data which can be more easily captured via linearity as opposed to trees (which are non-smooth piecewise constant functions). This is further supported by the observation that RF (AUROC 0.769) yielded a lower AUROC than RF+ (AUROC 0.781), a generalization of RF which models both smooth linear structure and nonlinear tree structure.

Remarkably, the variation in prediction accuracy across data preprocessing pipelines was substantially smaller than the variation in prediction accuracy across models. Figure 2(B) shows the range of mean AUROCs across the four data preprocessing pipelines for each method (Figure 2(B), left), compared to the difference between each method’s mean AUROC and that of the best-performing method (i.e., logistic regression with elastic net regularization) (Figure 2(B), right). Across all methods, the range of mean AUROCs across data preprocessing pipelines never exceeded 0.020 whereas the difference between logistic regression with elastic net regularization (the best-performing model) and RuleFit, GBDT, and FIGS were larger, exhibiting differences of 0.027, 0.027, and 0.109, respectively. These observations provide evidence that the trained models are not highly dependent on human choices made during the data preprocessing pipeline – a crucial stability check for fostering trust in our model development process.

Before proceeding to stage 2, we ultimately used this prediction check to filter out models with poor prediction performance, a possible indicator that the model does not accurately reflect reality and would generate unreliable interpretations.^6^ Here, we chose to use ordinary logistic regression as the “reference” model given its simplicity yet decent cross-validated prediction performance (AUROC 0.772) in this problem, and we dropped all models with worse prediction performance than logistic regression. Specifically, this prediction check excludes RuleFit, GBDT, and FIGS from the remainder of the analysis. Note that though RF (AUROC 0.769) has slightly lower prediction performance than logistic regression on average across the different data preprocessing pipelines, we did not exclude RF since at least one of its data preprocessing pipelines led to higher accuracy than that for logistic regression. In other words, the uncertainty due to data preprocessing is larger than the modeling difference between RF and logistic regression. We hence deemed that RF passed the prediction check and included RF in the remainder of the analysis.

### Stability-driven genes associated with high-grade prostate cancer

Having filtered out poor-performing prediction models and established that the prediction-checked models are indeed robust to different data preprocessing choices, we identified genes, which were both ranked highly important and highly stable across the four data preprocessing pipelines, six prediction-checked models, and ten development-test splits (i.e., 4 × 6 × 10 = 240 combinations), for use in the sMPS2 model.

#### Top-ranked genes across all data preprocessing pipelines and models.

**Figure 3.**
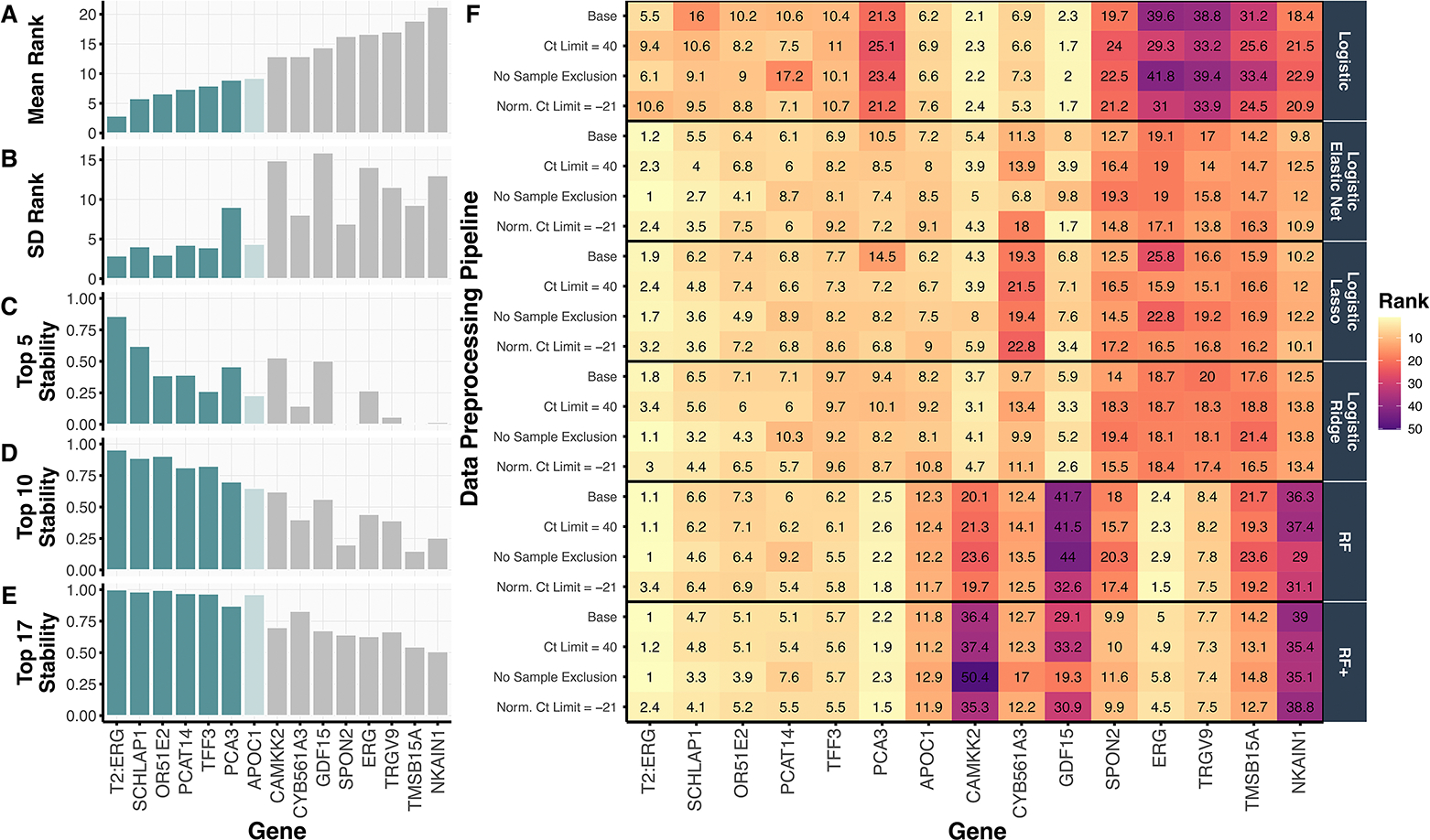
We summarize the top 15 ranked genes, according to **(A)** their mean gene ranking across four data preprocessing pipelines and six prediction-checked models, alongside **(B)** the variability of their gene rankings as measured by the standard deviation (SD) of this distribution and **(C-E)** the proportion of times that the gene appeared in the top 5, 10, and 17 genes. The top six genes were selected for the s^7^MPS2 model as they all achieved a low (i.e., highly important) mean rank (< 10) and good stability across the various metrics. The *APOC1* gene was also considered and used in the s^8^MPS2 model. **(F)** The heatmap shows a more granular view of the gene rankings, displaying the mean gene ranking per data preprocessing and model choice, averaged across 10 Development-Test splits. While the various data preprocessing and model choices generally yield similar gene rankings for the genes used in the simplified MPS models, logistic regression yields the most different gene rankings compared to the other models. In particular, it is the driving factor for *PCA3*’s large SD in (B).

In Figure 3(A), we show the mean ranking of each gene across the 240 preprocessing-model-split combinations alongside numerous stability metrics, including the standard deviation of each gene’s ranking (Figure 3(B)) and the proportion of times (out of 240) that the gene ranked in the top 5, 10, or 17 (out of 54) (Figure 3(C)–(E)). The top six-ranked genes *T2:ERG*, *SCHLAP1*, *OR51E2*, *TFF3*, *PCAT14*, and *PCA3* were all highly stable, each appearing in the top 10 ranked genes in more than 70% of the preprocessing-model-split combinations. The seventh-ranked gene, *APOC1*, also appears to be stably important; however, its stability declines when using only two of the four logistic-based regression models in the PCS-ensembled gene rankings (Figure S3). Notably, these top seven-ranked genes, selected from the 54 candidate genes under consideration, are a subset of the genes used in the validated MPS2 model.^5^

When examining the gene rankings per data preprocessing pipeline and method in Figure 3(F), we confirmed the robustness and stability of these trained models across data preprocessing choices, not only in terms of their resulting prediction accuracy as discussed in Section Prediction Check of Machine Learning Models via Cross Validation, but also in terms of their most important genes and model architecture. Figure 3(F) further reveals that the two genes *T2:ERG* and *PCA3* comprising the original MPS model were stably ranked as the top two most important genes according to RF and RF+ . Moreover, the top 6 genes were particularly stable across the regularized logistic regression models, RF, and RF+ fits while the ordinary logistic regression model generally produced a different set of gene rankings. Interestingly, the ordinary logistic regression model did not rank *PCA3* highly and is the main source of instability seen in the high SD for *PCA3* in Figure 3(B).

#### Top-ranked genes from specific models.

Beyond these top genes, Figure 3(F) illuminates several additional interesting gene ranking patterns across the different models and data preprocessing pipelines. First, there are genes, such as *CAMKK2* and *GDF15*, that tend to be more highly ranked when considering only linear structure (i.e., in the logistic-based models) while other genes, such as *ERG* and *TRGV9*, tend to be more highly ranked when allowing for nonlinear structures (i.e., in the tree-based models, RF and RF+). However, given that these prediction models yielded similar prediction accuracies, it’s unclear which model’s interpretation to trust over another. We hence selected the top 6 genes (*T2:ERG*, *SCHLAP1*, *OR51E2*, *TFF3*, *PCAT14*, and *PCA3*), together with the reference gene *KLK3*, for use in the final simplified 7-gene MPS2 model (s^7^MPS2), as these genes were highly stable across the various data-preprocessing, modeling, and Development-Test split combinations. Given the borderline stability status of *APOC1*, we also developed a simplified 8-gene MPS2 model (s^8^MPS2), which includes those genes in s^7^MPS2 along with *APOC1*.

### Internal assessment and validation of the sMPS2 models

**Figure 4.**
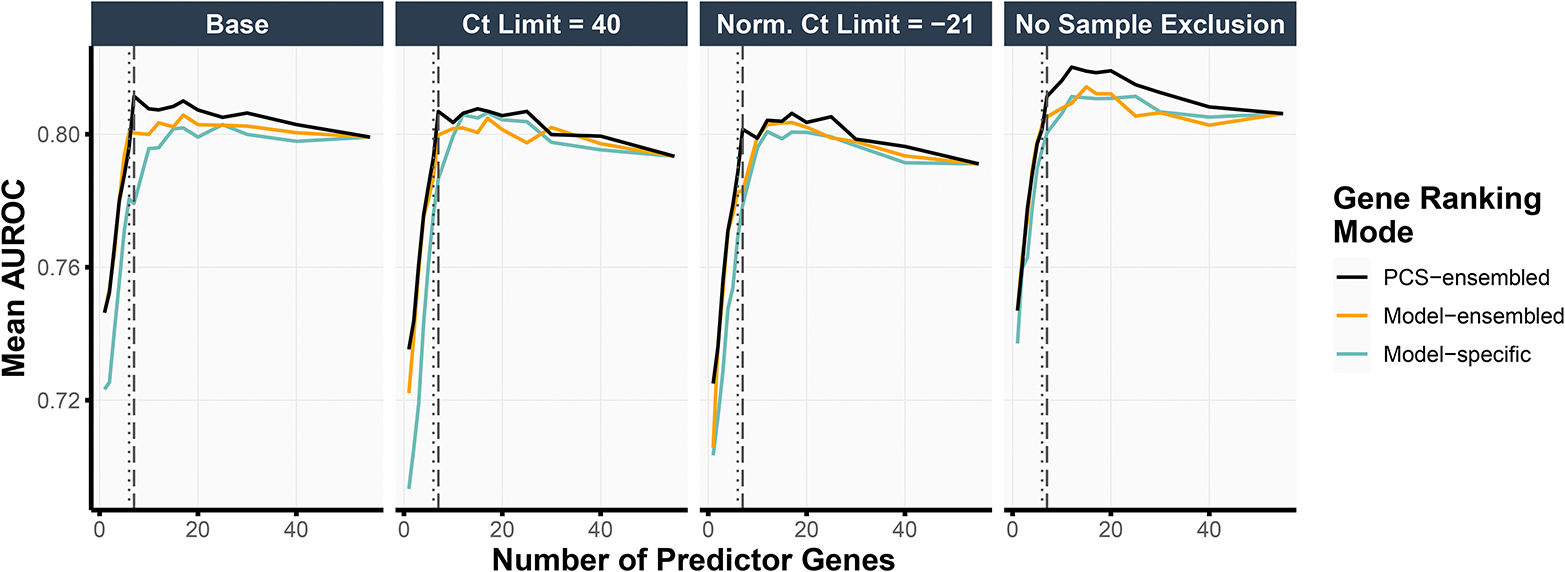
We show the mean AUROC, evaluated on the test set, when training the logistic regression model with ridge regularization using various choices of gene panel sizes (x-axis), data preprocessing pipelines (subplots), and gene rankings (color). The PCS-ensembled gene rankings yield the highest test AUROCs compared to other procedures for obtaining the gene rankings. Moreover, using 7 predictor genes (vertical dashed line) as in s^8^MPS2 gave the highest test AUROC in the base and Ct Limit = 40 data preprocessing pipelines and very competitive prediction performance in the remaining data preprocessing pipelines. Taking 6 predictor genes (vertical dotted line) as in s^7^MPS2 gave slightly lower but similarly competitive test prediction performance as 7 predictor genes.

Thus far, we have been primarily guided by the stability of the feature rankings when selecting the number of top-ranked genes in our final simplified MPS2 model. To assess the impact of this choice of the gene panel size (i.e., the number of top-ranked genes used in the model) on the prediction accuracy, we performed an internal validation assessment using the test set (details in Section Internal Validation for Evaluating Selected Genes). Figure 4 highlights the test prediction accuracies when using the logistic regression model with ridge regularization (other model results can be found in Figure S4). Taking the top 7 PCS-ensembled genes (as in s^8^MPS2) yielded the highest test AUROCs in the base and the Ct Limit = 40 data preprocessing pipelines (0.811 and 0.807, respectively) while also demonstrating competitive performance in the remaining two data preprocessing pipelines. The top 6 PCS-ensembled genes (as in s^7^MPS2) yielded similarly strong AUROCs, showing only a 0.01 drop in AUROC compared to taking the top 7 PCS-ensembled genes across all data preprocessing pipelines.

Moreover, like the cross-validation prediction accuracies (Section Prediction Check of Machine Learning Models via Cross Validation), these test prediction accuracies were very stable across the different data preprocessing choices. In particular, the AUROCs when taking the top 6 and 7 PCS-ensembled genes ranged between [0.788, 0.801] (difference of 0.013) and [0.801, 0.811] (difference of 0.010), respectively, across the different data preprocessing pipelines. When taking the top 17 genes using logistic regression with elastic net regularization as done in the original MPS2 model, the test prediction performance was also highly stable across data preprocessing pipelines, giving an AUROC range of [0.800, 0.811] (difference of 0.012). This demonstrates the robustness of both the sMPS2 models and the original MPS2 model against alternative data preprocessing choices and indicates that these potentially different, but equally-reasonable choices do not solely drive downstream conclusions.

We further assessed the impact of our stability-driven PCS-ensembled ranking approaches, which averages the gene rankings across both data preprocessing pipelines and models, as compared to alternative approaches – namely, a model-ensembled approach, which averages the gene rankings across models only, and a model-specific approach, which produces a unique gene ranking per model and data preprocessing choice (details in Appendix B.2). Across all data preprocessing pipelines and gene panel sizes, the top genes according to the PCS-ensembled gene rankings led to higher prediction accuracies than that from the model-ensembled or the model-specific gene rankings (Figure 4). This pattern also holds across different choices of prediction models (Figure S4).

### External cohort validation

**Figure 5.**
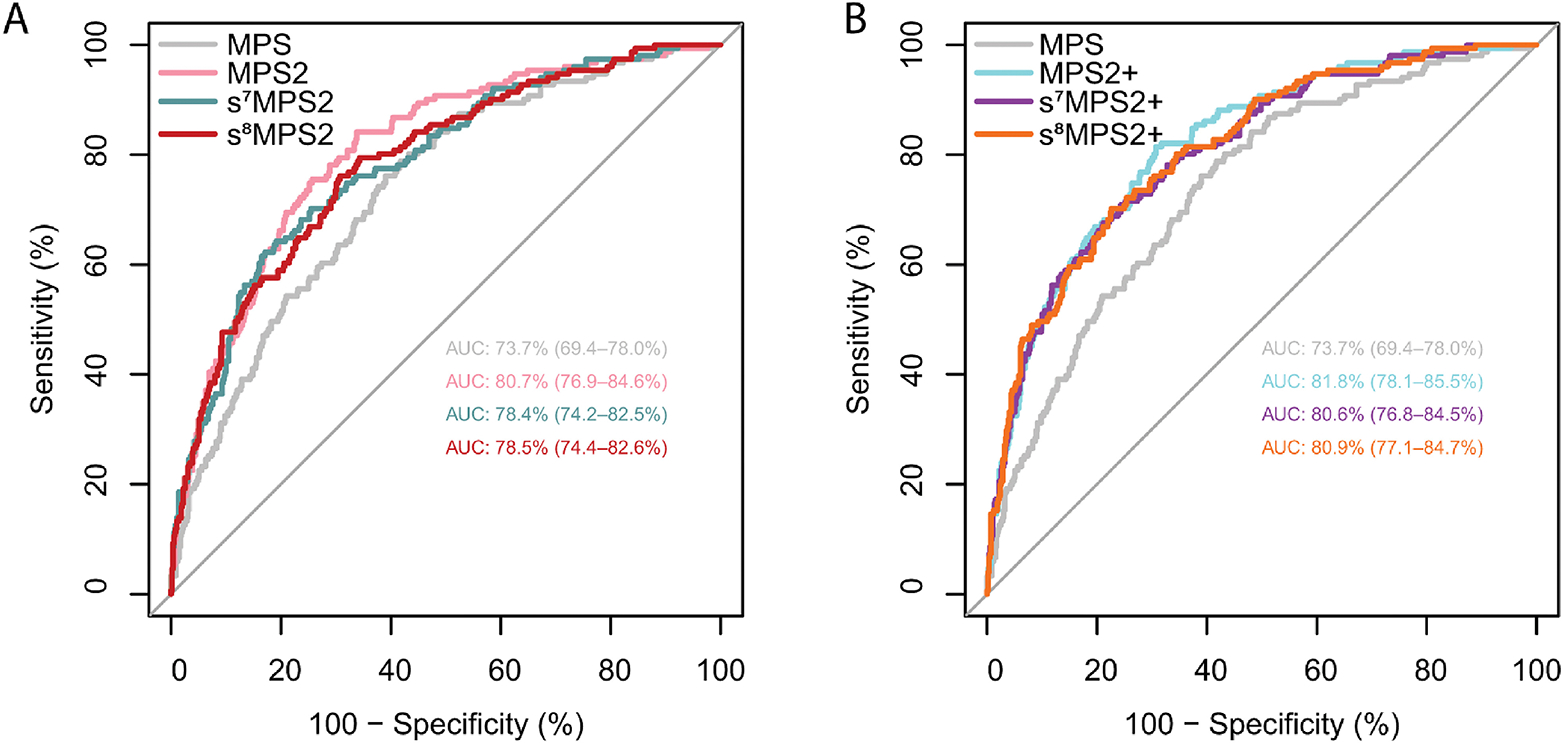
The AUROC curves from the blinded external validation cohort are shown for various urine-based biomarker tests without prostate volume (MPS, MPS2, s^7^MPS2, and s^8^MPS2, left) and with prostate volume (MPS2+, s^7^MPS2+, and s^8^MPS2+ right). The 7- and 8-gene simplified MPS2 models (s^7^MPS2/s^8^MPS2 and s^7^MPS2+/s^8^MPS2+) yield higher AUROCs compared to the 2-gene MPS model and competitive AUROCs compared to the 18-gene MPS2 model.

When evaluated on the NCI EDRN PCA3 Evaluation trial,^16^ the locked s^7^MPS2 model yielded an AUROC of 0.784 (95% confidence interval [CI], 0.742–0.825) for predicting high-grade PCa (Figure 5). This was a 4.7% improvement over MPS, which gave an AUROC of 0.737 (95% CI, 0.694–0.780), and only a 2.3% drop relative to the more complex 18-gene MPS2 model, which gave an AUROC of 0.807 (95% CI, 0.769–0.846). In the case when prostate volume is available, the s^7^MPS2 + model gave an AUROC of 0.806 (95% CI, 0.768–0.845), which was only a 1.2% drop relative to MPS2+ (AUROC: 0.818, 95% CI: 0.781–0.855). We also compared s^7^MPS2 to s^8^MPS2 in Figure 5, showing that the improvement when adding one additional gene (*APOC1*) is small. s^8^MPS2 and s^8^MPS2 + yielded AUROCs of 0.785 (95% CI, 0.744–0.826) and 0.809 (95% CI, 0.771–0.847), respectively. Importantly, the drops in AUROC (2.3%/2.2% for s^7^MPS2/s^8^MPS2 and 1.2%/0.9% for s^7^MPS2+/s^8^MPS2 + relative to MPS2 and MPS2+, respectively) are within the 1–2% uncertainty intervals induced by different data preprocessing choices. These AUROCs confirm the high diagnostic accuracy of the sMPS2 models as well as their similarly strong performance compared to the MPS2 models.

However, from a clinical perspective, it is also important to evaluate the performance of a practical clinical testing approach using a specific decision threshold that yields high sensitivity for high-grade PCa. In particular, since the traditional clinical approach of biopsying all men with elevated PSA is highly sensitive for PCa detection but leads to excess unnecessary biopsies, secondary tests such as biomarkers have focused on improving the specificity of screening while preserving the high sensitivity attained from a “biopsy all” approach. This emphasis on high sensitivity is reflected in current clinical guidelines, which propose the use of “biomarkers that improve the specificity of screening”.^31^ Considering the relative harms of false-negative and false-positive testing results and the proposed role of biomarkers for rule out testing (i.e., for ruling out the possibility of prostate cancer), we thus assessed thresholds providing 95% sensitivity for high-grade cancer. At this 95% sensitivity, s^7^MPS2/s^8^MPS2 provided a specificity of 32%/30%, a negative predictive value (NPV) of 96%/96%, and positive predictive value (PPV) of 26%/26% (Table 2). This corresponds to an estimated reduction of 318/297 unnecessary biopsies avoided per 1000 patients based upon the s^7^MPS2/s^8^MPS2 models. More interestingly, both s^7^MPS2 + and s^8^MPS2 + achieve similar, if not higher, specificity (40.7%), NPV (96.8%), and PPV (28.9%) than MPS2+ (40.5% specificity, 96.8% NPV, 28.9% PPV) and leads to an estimated 407 unnecessary biopsies avoided per 1000 patients under this clinical testing approach at 95% sensitivity. These estimates of the number of unnecessary biopsies are also complemented by a low false negative rate across all of the sMPS2 and MPS2 models. For each of these models, an estimated 52 biopsies would have been missed per 1000 patients at the 95% sensitivity level, had no other screening been undertaken. Detailed performance of the sMPS2 models at a range of high sensitivities with and without clinical factors are provided in Supplementary Table 1 and 2.

Finally, we evaluated the MTT^30^ to formally assess the tradeoff between the improvement in risk prediction and the additional cost of data collection between the 18-gene MPS2/MPS2 + and the smaller 7-gene s^7^MPS2/s^7^MPS2 + . At a 20% prevalence of high-grade prostate cancer (as seen in the validation cohort), the MTT is 229.5 and 122.0 for MPS2 + versus s^7^MPS2 + and MPS2 versus s^7^MPS2, respectively. Thus, at least 229 (or 122) sets of 18-gene MPS2+ (or MPS2) tests would be needed for every true positive to yield a positive net benefit relative to using s^7^MPS2+ (or s^7^MPS2). Furthermore, when incorporating the 1–2% uncertainty due to data preprocessing choices, the MTT ranges from [125.9, 1368.8] and [85.5, 214.7] for the s^7^MPS2 + and s^7^MPS2 tests, respectively. These large MTT ranges are a byproduct of the inverse decaying nature of MTT. Intuitively, as the prediction gap between the sMPS2 and MPS2 models decrease, the net benefit of using MPS2 over the sMPS2 models decreases rapidly and hyperbolically. Consequently, the small difference in AUROCs between the sMPS2 models and MPS2 after accounting for the uncertainty in data preprocessing translates to extremely large MTTs seen above. This tradeoff analysis suggests that MPS2 is not practically worthwhile from a data collection cost perspective, especially after accounting for the often-neglected uncertainty due to data cleaning, and further supports the accessibility and utility of the sMPS2 models for clinical care.

## Discussion

From a practical standpoint, it is preferable to have fewer variables in clinical assays, as this simplifies interpretation, reduces the potential for variation in measurements, and decreases costs. However due to the heterogeneity of prostate cancer, it may be necessary to increase the number of biomarkers to capture different forms of aggressive prostate. For example, the recurrent *T2:ERG* fusion of prostate cancer occurs in just about half of the patients. With exhaustive screening previously done^5^ and extensive stability testing across various perturbation situations in this study, we identified the most stable and important biomarkers that contribute to prediction of clinically significant prostate cancer. Unsurprisingly, all of the top 6 most stable genes identified by the PCS ranking (*T2:ERG*, *PCA3*, *SCHLAP1*, *OR51E2*, *TFF3* and *PCAT14*) have been reported to be overexpressed in prostate cancer and their roles as biomarkers have been explored by independent studies.^32–39^ The additional gene included in the s^8^MPS2, *APOC1* (apolipoprotein C1), is also frequently reported to play a role in various cancer types including breast, ovarian, pancreatic, gastric cancers and be associated with tumor infiltrated macrophage.^40–44^ It is further worth noting that the genes used in the s^7^MPS2/s^8^MPS2 models were selected out of 54 candidate genes and yet are a subset of the 18 genes used in the validated MPS2 model,^5^ reinforcing their potential biological functions and prognostic values. Moreover, the resultant simplified MPS2 models built upon the aforementioned genes (s^7^MPS2/s^8^MPS2) achieved comparable performance as the original models on the external validation cohort (AUROC 0.784/0.785 vs 0.807) (within the uncertainty size of 1–2% from making different but reasonable data preprocessing choices); for the models with prostate volume, the difference became even smaller (AUROC 0.806/0.809 vs 0.818) (Figure 5). At the clinically relevant sensitivity of 95%, s^7^MPS2/s^8^MPS2 was parallel to the original model with about 96% NPV though the specificity decreased from 37% to 32%/30% while s^7^MPS2+/s^8^MPS2 + was equivalent to the original model in both NPV and specificity (Table 2). These findings, combined with the MTT analysis, suggest that clinical use of the simplified MPS2 models would benefit patients to a similar extent as the 18-biomarker MPS2, avoiding unnecessary biopsy while maintaining high sensitivity for high-grade cancers from early detection, at a substantially lower data collection cost.

From a methodological perspective, we demonstrate the value of the PCS framework for stress-testing various human judgment calls throughout the model development process. To facilitate both transparency and reproducibility, we provide extensive documentation and justification of our data preprocessing and model development process in the supplementary PCS documentation alongside all code on GitHub (https://github.com/Yu-Group/sMPS2) to reproduce our analysis. This work not only sheds light on such decisions in an effort towards more transparent and truthful science, but ensures that these different but equally-reasonable choices do not drastically impact downstream conclusions, fostering greater trust in the model. Going beyond this rigorous stress-testing, the PCS framework can also enhance the model itself. Here, we exploit the stability of similarly predictive models in order to simplify an 18-gene model into a 7-gene (or 8-gene) model for predicting high-grade PCa without substantially reducing its accuracy. Our overarching PCS ranking pipeline is more generally applicable, and as exemplified by the strong and generalizable prediction performance of the sMPS2 models, opens the door to more rigorous and trustworthy development of robust, highly-interpretable biomarker tests.

This study has some limitations. First the patient cohorts in this study were not suitable for comparing with MRI screening or evaluating the clinical benefit of combining MRI with biomarkers for screening. Due to the proposed use of the sMPS2 assay as a pre-mpMRI, prebiopsy test to rule out the need for mpMRI or biopsy, we excluded men with a history of prostate mpMRI and targeted biopsy. Second, although the total sample size of the training and testing cohorts are adequate, larger sample sizes are needed to evaluate the performance of sMPS2 in specific subpopulations, for example, African American, repeat biopsy population, and patients of non ETS fusion molecular subtype. Additionally, the performance metrics reported in this study are based on 95% sensitivity and 20% high-grade prevalence in the validation cohort (which is within reported range from 17% to 31%), representing a single data point to illustrate the clinical applicability. However, given that predictive values are affected by prevalence, the high-grade prevalence of the target population may vary from institute to institute and change over time as more intervention strategies come into play, re-calibrating or updating the model is necessary to improve the performance. Lastly, given the complex correlation structure of the data, we cannot guarantee that the identified 7 (or 8) genes used in the sMPS2 models are the best set of 7 (or 8) genes that lead to the highest prediction accuracy. Nonetheless, the current sMPS2 models demonstrate excellent robustness and generalizability at a reduced data collection cost compared to existing models, underscoring its clinical relevance and applicability.

## Supplementary Material

Appendices A and B

Supplementary Table 2

Supplementary Table 1

Supplemental material for this article is available online.

## Figures and Tables

**Table 1.** Prediction methods, software implementations, and hyperparameters under study.

Prediction Method	Implementation	Hyperparameters*

Logistic regression	‘LogisticRegression()’ in sklearn python package	No hyperparameters
Logistic regression with *L*_*1*_ (Lasso) regularization	‘LogisticRegression(penalty=“11”)’ in sklearn python package	C = 10^i^ for i = −3, −2.5, 2, ..., 2, 2.5, 3
Logistic regression with *L*_*2*_ (ridge) regularization	‘LogisticRegression(penalty=“12”)’ in sklearn python package	C = 10^i^ for i = −3, −2.5, 2, ..., 2, 2.5, 3
Logistic regression with combined *L_1_* + *L_2_* (elastic net) regularization		
‘Logistic Regression (penalty=“elasticnet”)’ in sklearn python package	C = 10^i^ for i = −3, −2.5, 2, 2, 2.5, 3 11_ratio = 0.1, 0.25, 0.5, 0.75, 0.9	
Random forest	‘RandomForestClassifier()’ in sklearn python package	min_samples_leaf = 1, 3, 5, 10 n_estimators = 500
Gradient boosting decision trees	‘GradientBoostingClassifier()’ in sklearn python package	learning rate = 0.05, 0.1, 0.15, min_samples_leaf = 1, 5, 10 max_depth = 3, 5 n estimators = 500
RuleFit	‘RuleFitClassifier()’ in imodels python package	max_rules = 5, 10, 30, 50
Random forest+	‘RandomForestPlusClassifier()’ in imodels python package	Default hyperparameter grid used
Fast interpretable greedy-tree sums	‘FIGSCIassifier()’ in imodels python package	max_rules = 5, 10, 12, 15, 20, 30, 50

* Hyperparameters were tuned using 5-fold cross-validation. Unless specified, the default hyperparameters for each method were used.

**Table 2.** Performance of MPS2, MPS2+and corresponding sMPS2 models (7- and 8-biomarkers) in the validation cohort at the 95% sensitivity level.

Model	Sensitivity (%)	Specificity (%) (TN/(TN + FP))	Negative Predictive Value (%) (TN/(TN + FN))	Positive Predictive Value (%) (TP/(TP + FP))	Estimated unnecessary biopsies avoided per 1000 patients

MPS	95.0	23.0 (136/592)	94.4 (134/144)	23.9 (143/599)	230
MPS2	95.0	37.0 (219/592)	96.5 (219/227)	27.7 (143/516)	370
s^7^MPS2	95.0	31.8 (188/592)	95.9 (188/196)	26.1 (143/547)	318
s^8^MPS2	95.0	29.7 (176/592)	95.7 (176/184)	25.6 (143/559)	297
MPS2+	95.0	40.5 (240/592)	96.8 (240/248)	28.9 (143/495)	405
s^7^MPS2+	95.0	40.7 (241/592)	96.8 (241/249)	28.9 (143/494)	407
s^8^MPS2+	95.0	40.7 (241/592)	96.8 (241/249)	28.9 (143/494)	407

TN=true negatives; FP = false positives; FN = false negatives; TP = true positives

## Data Availability

As the data represented in this publication are from a blinded NCI-EDRN validation cohort, unblinding of the data is not allowed. To maintain patient confidentiality, no individual level data will be provided, only patient summaries. All code to reproduce this analysis can be found on GitHub: https://github.com/Yu-Group/sMPS2.
