# Supplementary material for: A simplified MyProstateScore2.0 for high-grade prostate cancer
Source: Cancer Biomark. Author manuscript; Available in PMC 2025 Jun 4. (PMC12135703; doi:10.1177/18758592241308755)
Supplement: Supplementary file 1 [file NIHMS2076637-supplement-Appendices_A_and_B.pdf]

Appendix A: Data Preprocessing of Gene Expression Data Details

We first preprocessed the gene expression data by following a similar data preprocessing procedure as taken in the original development of MPS2.[5] In this procedure, we first set the upper Ct value limit to 35. Specifically, Ct values greater than this limit were considered undetected and set to 35. Ct values from OpenArray™ that were "Undetermined" or "Inconclusive/No Amp" were also considered to be undetected and set to the upper Ct value limit of 35. We chose this value of 35 as opposed to the commonly used 40 since previous work showed that setting the Ct value limit to 40 can often introduce unwanted biases and that setting this limit to 35 can effectively reduce this bias for non-detects in qPCR.[45] Next, we computed the standard deviation (SD) across 3 technical replicates. If SD >= 1, the replicate farthest from the mean was removed; otherwise, all 3 replicates were kept. The average Ct value across these kept technical replicates was then calculated. As a filter for poor quality samples, all samples with an average Ct value of the reference gene *KLK3* above the 95th percentile were excluded. We next normalized the average Ct values for each target gene by *KLK3* using the formula -[average Ct of gene X - average Ct of *KLK3*]. Finally, z-score scaling was applied to the normalized average Ct before downstream model development and feature selection. We refer to this data preprocessing pipeline as the *base preprocessing pipeline*.

While the base preprocessing pipeline serves as a reasonable starting point, it is important to note that human judgment calls or choices such as the procedure for handling undetectable Ct values and poor quality samples were inevitably made. Hence, to improve the robustness of our model and conclusions against these preprocessing choices, we examined three alternative data preprocessing pipelines, each equally-reasonable but a slight modification of the base preprocessing pipeline.

1. **Ct limit = 40 preprocessing pipeline:** Rather than setting the upper Ct value limit to 35 for undetected replicates, we instead set the upper Ct value limit to 40. All other preprocessing steps remain unchanged from the base preprocessing pipeline.
2. **Normalized Ct limit = -21 preprocessing pipeline:** In the aforementioned data preprocessing pipelines, the Ct values for undetected replicates were set prior to the normalization of the Ct values. Thus, the normalized Ct value for undetected replicates differs between genes. For comparison, in this preprocessing pipeline, we instead replace the Ct values for all undetected replicates after Ct normalization to have a constant value of −21 (which was the lowest Ct value post-normalization). All other preprocessing steps remain unchanged from the base preprocessing pipeline.
3. **No sample exclusion preprocessing pipeline:** Rather than excluding all samples with an average Ct value of the reference gene *KLK3* above the 95th percentile, this preprocessing pipeline does not exclude any samples based upon their Ct value for the reference gene *KLK3*. All other data preprocessing steps remain unchanged from the base preprocessing pipeline.

While we focus our attention on the four described data preprocessing pipelines (i.e., the base preprocessing pipeline and three alternative preprocessing pipelines above) due to computational constraints, we acknowledge that these preprocessing pipelines encompass only a small fraction of all possible data preprocessing choices. Still, we believe that assessing model robustness and stability across these different preprocessing pipelines provides a valuable step towards scientific findings that are not solely reliant on particular (reasonable) data preprocessing decisions. In the spirit of transparency, we provide extensive documentation of our data preprocessing decisions and justify them when possible in the supplementary PCS documentation on GitHub (https://github.com/Yu-Group/sMPS2).

Appendix B: Model Development Details

B1. Prediction Check

In the first stage of our model development process, we conducted a prediction check, where we assessed model prediction performance in order to filter out models which may not accurately represent the scientific phenomena under study as suggested by their poor prediction performance.[6] Specifically, we first randomly split the Development Cohort (Section Development Cohort) into a Development (80%) and Test Set (20%). Then using the Development Set, we performed 4-fold cross-validation (CV). For each CV fold, we trained each model (described in Section Modeling Choices) on each of the four preprocessed datasets (described in Section Data Preprocessing of Gene Expression Data and Appendix A) using the training fold and evaluated the prediction error on the validation fold. The covariates used included the 54 nominated genes from the original MPS2 study and available clinical variables that are generally thought to be associated with high-grade PCa (age, race, family history of prostate cancer, abnormal DRE, prior negative biopsy, and PSA).[17] The cross-validation (CV) error was computed using three different evaluation metrics - area under the receiver operating characteristic curve (AUROC), area under the precision recall curve (AUPRC), and classification accuracy - and averaged across the four CV folds. This process was repeated for 10 different Development-Test splits. Models that did not outperform ordinary logistic regression in any of the data preprocessing pipelines across the three evaluation metrics were excluded from further analysis and failed the prediction check.

B2. Stability-driven Gene Ranking

For those models that passed the prediction check, we proceeded to compute a ranking of gene (or feature) importances for each preprocessing-model combination. More precisely, for each data preprocessing pipeline and model specification, we computed a measure of feature importance from the model fit on each CV training fold, averaged the feature importance measures across the 4 folds, and ranked the features according to this averaged feature importance for the given method. For random forest (RF) and random forest+ (RF+), we respectively used mean decrease in impurity (MDI)[21] and mean decrease in impurity+ (MDI+)[24] as the feature importance measures. For logistic regression and logistic regression with L_2 (ridge) regularization, we measured feature importance using the magnitude of the estimated regression coefficients. For logistic regression with L1 (Lasso) or the combined L1 + L2 (elastic net) regularization, we measured feature importance by the number of times each feature had a non-zero coefficient across the 4 CV folds; if there are ties, we broke these ties based upon the magnitude of the estimated regression coefficients. Note that the covariate data was centered and scaled to have mean 0 and variance 1 prior to fitting these models. Additionally, while clinical variables were used as covariates in the trained models (and included in the feature importance computation), these clinical variables were dropped when computing the feature importance rankings since our aim is to identify the most important genes. Thus far, the aggregation of feature importances and resulting feature importance ranking is model-specific, i.e., each model or method yields its own feature importance ranking.

However, among the models that passed the prediction check and yielded fairly similar prediction performance, it is unclear whether or why one should trust the feature importance rankings from one method over another. Following the stability principle of the PCS framework,6 we hence examined the features that were most stably important across these similarly-performing prediction models. To do so, we averaged the model-specific feature ranks across models, yielding a model-ensembled feature importance ranking, and across both data preprocessing pipelines and models, yielding a PCS-ensembled feature importance ranking. Note that this stability-driven feature ranking is performed per Development-Test split, using the same splits as that in the prediction check (Table S1).

B3. Selection of Stable Genes

After obtaining the various gene rankings for each Development-Test split, we examined the stability of the top-ranked genes across the different Development-Test splits via various metrics (applying the stability principle in PCS to metric choices) in order to identify the sparse set of stable genes used in the final simplified MPS2 model. The first stability metric was the proportion of times each gene was ranked in the top k across all preprocessing-model specifications and Development-Test splits (4 data preprocessing pipelines × 6 models that passed the prediction check × 10 splits = 240 total configurations). Here, we tried several different choices of k: $k = 5$ and 10 to identify genes that were almost always the most important (~top 10% or 20%) out of the genes under consideration, and $k = 17$ for comparison with the original MPS2 study.[5] The second stability metric was the average PCS-ensembled feature importance ranking across all Development-Test splits. Note that this is equivalent to the average feature importance ranking across all data preprocessing pipelines, models, and Development-Test splits. The third stability metric is the standard deviation (SD) of the gene's importance rankings across all data preprocessing pipelines, models, and Development-Test splits. Using these various stability metrics, we identified the genes which were frequently ranked in the top 5, 10, and 17 features, had a high average feature ranking (i.e., ranked close to 1), and whose feature rank was highly stable (or had low SD or variability) across the different preprocessing pipelines, models, and Development-Test splits. We acknowledge that a heuristic approach, combined with expert domain knowledge, guided our final selection of genes and that no formal pre-specified thresholds were used for selection. However, these selected genes were locked prior to internal and external validation, and thus, these validation studies (detailed in Sections Internal Validation for Evaluating Selected Genes and Model Validation on Blinded, External Cohort) provide a proper, unbiased assessment of our model's generalizability.
